# Eyelid heat pain sensitivity in healthy participants

**DOI:** 10.1097/PR9.0000000000001337

**Published:** 2025-09-22

**Authors:** Nicholas J. Pondelis, Vanda Faria, Hailey Krasnikov, Cameron Talbert, Madison Dudek, Paula A. Sepulveda-Beltran, David Valdes-Arias, David Zurakowski, Anat Galor, Scott Holmes, Elizabeth R. Felix, Eric A. Moulton

**Affiliations:** a Brain and Eye Pain Imaging Lab, Pain and Affective Neuroscience Center, Department of Anesthesiology, Critical Care and Pain Medicine, Boston Children's Hospital and Harvard Medical School, Boston, MA, USA; b Pediatric Pain Pathways Lab, Pain and Affective Neuroscience Center, Department of Anesthesiology, Critical Care and Pain Medicine, Boston Children's Hospital and Harvard Medical School, Boston, MA, USA; c Bascom Palmer Eye Institute, University of Miami Miller School of Medicine, Miami, FL, USA; d Department of Anesthesiology, Critical Care and Pain Medicine, Boston Children's Hospital and Harvard Medical School, Boston, MA, USA; e Research Service, Miami Veterans Administration Medical Center, Miami, FL, USA; f Department of Physical Medicine and Rehabilitation, University of Miami Miller School of Medicine, Miami, FL, USA; g Department of Ophthalmology, Boston Children's Hospital and Harvard Medical School, Boston, MA, USA

**Keywords:** Ophthalmology, Nociception, Ocular, Threshold, Psychophysiology, QST

## Abstract

**Introduction:**

Chronic ocular pain (COP) is a widespread pain condition that presents clinical challenges, has heterologous and overlapping etiologies across patients, and can have a debilitating effect on quality of life. It can manifest with neuropathic-like symptoms as well as dysesthesias associated with afferent disruption, brain dysfunction, or both, complicating diagnosis and treatment. Quantitative sensory testing (QST) has been used extensively in individuals with chronic pain to detect abnormal peripheral and central nervous system function. Investigating relevant sensory thresholds is crucial for integrating QST into ocular pain assessments.

**Objectives:**

This study aimed to determine heat pain thresholds on the eyelid in healthy participants. Furthermore, we sought to evaluate the eyelid as a feasible and suitable body site for COP investigation using QST.

**Methods:**

Heat pain thresholds were measured in 80 healthy participants at 2 study sites using the method of limits at 3 stimulation sites: the upper eyelid, the forehead, and the volar forearm.

**Results:**

Analysis revealed a significantly lower estimated marginal mean (EMM) heat pain threshold on the eyelid (42.5 ± 0.4 EMM ± SE) compared with the forehead (46.6 ± 0.4 EMM ± SE) (*P* < 0.001) and forearm (46.4 ± 0.4 EMM ± SE) (*P* < 0.001). These differences were statistically significant regardless of sex, study site, or age. No differences were found between the forehead and the forearm (*P* = 1.00).

**Conclusion:**

The eyelid is a clinically relevant site for QST in the investigation of ocular and trigeminal function, and this study sets the groundwork for applying eyelid QST in future research of ocular pain and disease etiology.

## Abstract 2

Supplemental Digital Content is Available in the Text.

Quantitative sensory testing was performed to assess heat pain thresholds on the upper eyelid. Eyelid thresholds were significantly lower than the forehead or forearm.

## 1. Introduction

Chronic ocular pain (COP) is a prevalent condition that frequently leads to eyecare visits, increases health care costs, and profoundly affects quality of life.^20,21,44^ Chronic ocular pain can be challenging to diagnose and treat as its presence can be driven by various etiologies including dry eye disease, anatomical abnormalities, and neurosensory dysfunction, among many others.^9,56^ In fact, neuropathic mechanisms contribute to COP in some individuals, with spontaneous pain often described as burning and accompanied by heightened sensitivity to light and wind (ie, allodynia and hyperalgesia). These symptoms can be driven by peripheral receptor abnormalities, disruptions in afferent pathways, alterations in cortical and subcortical brain processing, or a combination of these factors.^5,11,57^ Although diagnosis and understanding of COP contributors has improved over the past 2 decades, a better characterization of peripheral and central contributions to somatosensory function would help elucidate underlying causes, differentiate pain subtypes (nociceptive vs neuropathic), and improve treatment strategies.^6,31,58^

One method used to interrogate peripheral and central pain mechanisms is quantitative sensory testing (QST).^25,65^ QST can help detect changes in sensory nerve pathways, infer underlying mechanisms, and potentially aid in symptom management and the prediction of treatment efficacy.^2,16,42,67^ Thus, QST may offer insight into painful ocular conditions and the integrity of the trigeminal system.^14,55,62^To classify and understand the pathophysiology of chronic and neuropathic pain conditions, it is essential to distinguish abnormal sensory responses (indicating nerve dysfunction) from healthy reference values (indicating intact nociception).^41,54,60^

Historically, ocular QST investigations applied heated probes to the cornea with contradictory results; some participants reported no thermal sensations, while others were unable to complete testing because of excessive apprehension.^38,47^ Thus, modern studies of ophthalmic conditions using thermal QST have been restricted to nonocular sites when using direct contact testing^22,69^ or have used noncontact protocols targeting the cornea.^1^ Noncontact testing has many advantages; however, it requires specialized equipment and some modalities (eg, laser stimulation) merit extra concern when used near the eye.^1,4,66^ Given the challenges surrounding direct contact corneal testing, and the specialized equipment required for noncontact testing, the eyelid emerges as a feasible and valid test site for investigations of thermal sensitivity that may be generalized to the eye.

Only 2 reports have documented eyelid temperature sensations, and both primarily used the eyelid as a methodologic control rather than an experimental end point.^7,47^ One, from 1937, describes indifference to 33°C stimuli, but that 51.5°C stimuli elicited pain described as “intensively rather severe”.^47^ The other, published in 1979, used a water jet immersion system.^7^ Hence, no thermal testing has been performed on the eyelid using commercially available QST devices.^68^ To bridge this knowledge gap, we quantified heat pain thresholds (HPTs) on the eyelid, forehead, and forearm in healthy participants. Establishing and reporting sensory threshold data for this eye structure is a crucial step in the process of integrating QST into the investigation of ocular pain phenotypes and thus in the advancement of COP diagnostic capability and the development of precision therapies for ocular pain conditions.

## 2. Methods

### 2.1. Participants

The Boston Children's Hospital (BCH) Institutional Review Board (IRB) approved all aspects of this research and is the IRB of record for the two-site study involving BCH and the University of Miami (UMiami). The study met the guidelines for ethical human research set by the Helsinki Accord.

Healthy, pain-free participants were recruited from IRB-approved advertisements, flyers, and brochures placed at approved hospital sites, community-based centers and bulletin boards, web advertisements, local community listservs and hospital research announcements, as well as word of mouth.

Before consenting to the study, all participants were screened for inclusion and exclusion criteria. Eligible participants met inclusion criteria of ≥10 years old; medications stable for ≥4 weeks; and pain-free. Exclusion criteria included history of dry eye disease, chronic ocular pain, or photoallodynia (ie, photophobia); severe ocular conditions (corneal abnormalities, glaucoma, hard contact lens use, retinal abnormalities, scleritis, uveitis, history of corneal surgery, and history of allergic reaction to any eye drop); chronic medical conditions that feature pain (arthritis, deep vein thrombosis, endometriosis, low-back pain, and tendonitis); neurological disease (amyotrophic lateral sclerosis, Alzheimer's, brain tumor, cerebrovascular accident, seizure disorders, idiopathic hypersomnia, migraine, multiple sclerosis, neuropathy, Parkinson's, severe traumatic brain injury, severe mental illness [DSM-5 axis I psychiatric disorder except anxiety and depression], and spinal injury); inherited disorders associated with ocular manifestations; and medical conditions with potential ocular manifestations (asthma, diabetes, hypertension, HIV, sarcoidosis, and thyroid disease). These criteria were evaluated based on self-report. Participants who met criteria were considered healthy for the purposes of this study. All participants provided written informed consent or assent before study procedures began.

### 2.2. Quantitative sensory testing protocol

Heat pain thresholds were measured using a TSA2 Advanced Thermosensory Stimulator with a 6-mm diameter (28.3-mm^2^ stimulus area) Peltier thermode (Medoc Ltd., Israel). All participants received consistent scripted verbal instructions based on those published from the German Research Network on Neuropathic Pain^54^ and previous studies in our laboratory,^14,22^ and adapted for this protocol (see supplementary methods, supplemental digital content, http://links.lww.com/PR9/A344).

**Figure 1.**
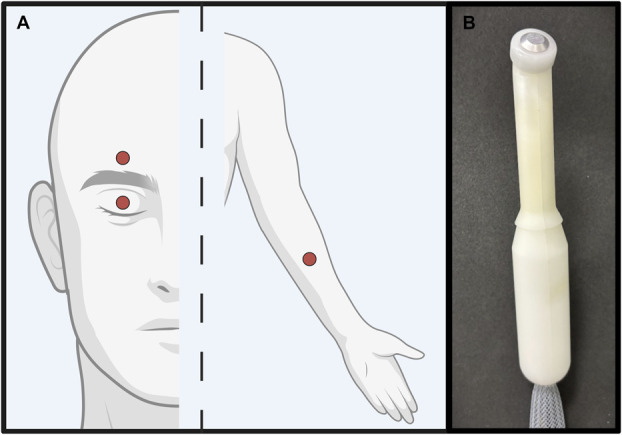
(A) Stimulation sites: eyelid, forehead, and forearm. (B) 6-mm Peltier thermode (Medoc Ltd., Israel). Created in BioRender: Pondelis, N. (2025) https://BioRender.com/h89m112.

Heat pain thresholds were measured using the method of limits with an ascending series at 3 stimulation sites: the right forehead, above the eyebrow aligned with the pupil; the left volar forearm, midway between the wrist and cubital fossa; and the center of the right upper eyelid (Fig. 1). Heat pain thresholds were assessed in the order of a forehead trial, then a forearm trial, then an eyelid trial; this pattern constituted one sequence. This sequence was repeated 3 times, with at least a 15-second interstimulus interval between each trial, and a 45-second interval between each sequence. This ensured that each stimulation site would have less than 90 seconds between repeated stimuli. Participants were instructed to close their eyes before each stimulus for consistency between stimulation sites and to promote focused attention on the sensation. Stimulation sites were marked before testing began using a highlighter to ensure consistent thermode placement across trials.

The thermode baseline temperature was set to 32°C, and the thermode was placed on the skin site at least 6 seconds before the trial to allow the skin to acclimate to the baseline temperature. Upon starting the trial, the thermode temperature increased by 1°C/s. Participants were instructed to press a stop button at the first moment they experienced pain, when the sensation changed “in quality from ‘hot’ to, for example, ‘burning’ or ‘stinging’ hot.” If the participant did not press the response button before the thermode reached 50.1°C, the device would automatically terminate the trial and the thermode would return to baseline temperature.

Immediately after pressing the button to indicate the presence of pain sensation, the thermode was removed and participants were presented with 2 computerized visual analogue scales (VAS). The first VAS was labeled pain intensity with “no pain at all” on the left side of the scale and “the most intense pain imaginable” on the right. The second VAS was labeled unpleasantness with “not unpleasant at all” on the left side and “the most unpleasant sensation imaginable” on the right. Participants were asked to adjust computerized sliders on each VAS to rate the pain intensity and unpleasantness of the sensation they felt at the time they pressed the button. Visual analogue scale ratings were made after every trial. Before the first trial began, a practice trial was conducted on the right hand to familiarize participants with the protocol before data collection.

Visual analogue scale ratings of pain intensity and unpleasantness of the sensations were scaled to 0 to 100 for quantification, with higher numbers indicating more severe sensations.

Study data were collected and managed using REDCap (Research Electronic Data Capture), a secure, web-based software platform designed to support data capture for research studies, hosted at BCH.^28,29^

## 3. Data analysis

Heat pain thresholds and VAS ratings were calculated for each stimulation site by taking the average across the 3 trials.

Three repeated-measures linear mixed model (LMM) main effect analyses were performed with mean data to assess the factors of stimulation site (eyelid, forehead, and forearm), sex (male vs female), and study site (BCH vs UMiami) on HPT and VAS ratings. Age was included as a continuous covariate in the models and used to derive estimated marginal means (EMM). Post hoc pairwise comparisons were performed, and significance values were adjusted by the Bonferroni correction to account for multiple test comparisons.

Heat pain threshold, pain intensity ratings, and unpleasantness ratings are reported as mean ± standard deviation (SD) and median (interquartile range) for raw values and mean ± standard error (SE) for EMM. Linear mixed model age covariance results are reported as a parameter estimate (PE) ± SE for each LMM analysis.

A *P* value of less than 0.05 was considered statistically significant.

Statistical analyses were performed using SPSS software ver. 29 (IBM, Armonk, NY).

## 4. Results

### 4.1. Participant demographics

A total of 97 individuals were recruited. Seventeen did not qualify as healthy and were excluded from analysis. Participants included 80 individuals age 10 to 59 years, with a mean age of 25.1 ± 10.8 years. Fifty percent of participants were assigned female at birth, 63% self-identified as White, and 18% reported being Hispanic (Table 1). The BCH study site enrolled 64 participants, and the UMiami study site enrolled 16 participants. Comorbidities and medications are presented in the supplemental digital content (see supplementary table 1, http://links.lww.com/PR9/A344).

**Table 1:** Demographics.

Descriptive characteristics	n (%)
Sex assigned at birth	
Male*	40 (50%)
Female	40 (50%)
Race†	
American Indian/Alaska Native	2 (3%)
Asian	17 (21%)
Black or African American	8 (10%)
White	50 (63%)
Something else	7 (9%)
Ethnicity	
Hispanic	18 (23%)
Non-Hispanic	62 (78%)

* One participant identified as nonbinary but was assigned male at birth.

† Four participants self-identified with multiple race categories.

### 4.2. Heat pain thresholds for the eyelid, forehead, and forearm

**Figure 2.**
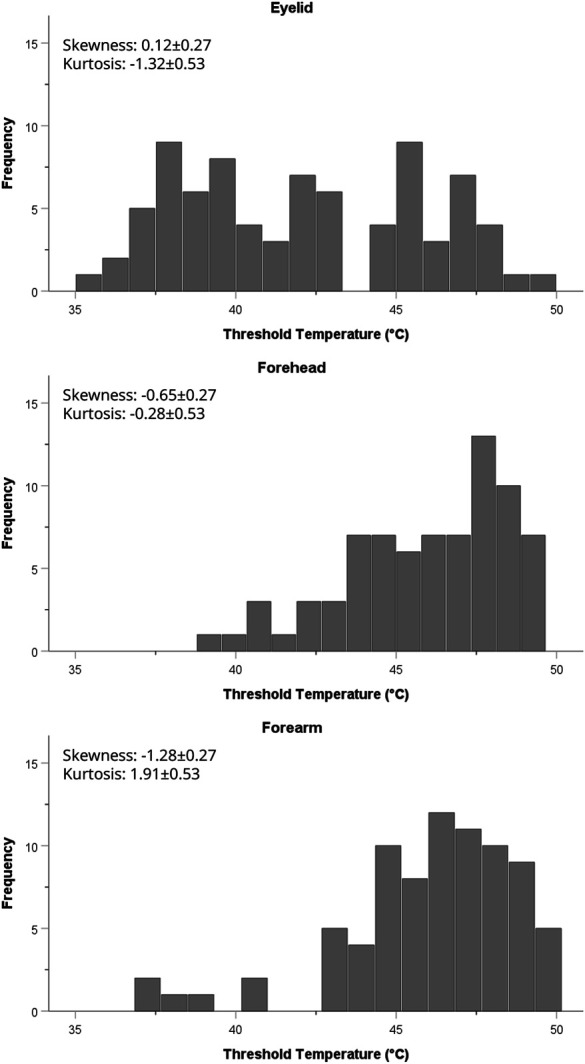
The distribution of HPTs across participants for the eyelid, forehead, and forearm. Skewness and kurtosis presented as statistic ± standard error.

The distributions of HPTs across participants for each stimulation site are depicted in Figure 2. Eyelid HPTs were mildly positively skewed (0.12 ± 0.27) and moderately platykurtic (−1.32 ± 0.53), and display a bimodal distribution. Forehead HPTs were moderately negatively skewed (−0.65 ± 0.27) and mildly platykurtic (−0.28 ± 0.53). Forearm HPTs were severely negatively skewed (−1.28 ± 0.27) and moderately leptokurtic (1.91 ± 0.53).

Mean and median HPT data for each stimulation site were averaged across subjects. Estimated marginal means were calculated to adjust for the continuous age covariate. Age was evaluated at the mean of 25.1 years for main effect analyses to ensure factor analyses were not confounded by age differences (Table 2).

**Table 2:** Raw means, estimated marginal means with 95% confidence intervals, and medians for HPT, pain intensity, and unpleasantness for each stimulation site.

	Eyelid	Forehead	Forearm
HPT (°C)			
Raw mean ± SD	42.1 ± 3.8	46.2 ± 2.6	46.0 ± 2.8
EMM ± SE	42.5 ± 0.4*	46.6 ± 0.4	46.4 ± 0.4
EMM 95% CI	41.7–43.3	45.8–47.5	45.6–47.3
Median (IQR)	42.1 (38.8–45.5)	46.7 (44.4–48.3)	46.5 (44.7–48.0)
Pain intensity (0–100)			
Raw mean ± SD	23.4 ± 21.3	24.2 ± 21.3	26.3 ± 22.0
EMM ± SE	24.0 ± 3.1	24.8 ± 3.1	26.9 ± 3.1†‡
EMM 95% CI	17.9–30.1	18.7–31.0	20.8–33.0
Median (IQR)	18.3 (6.1–32.8)	16.5 (7.7–36.8)	22.2 (8.9–37.0)
Unpleasantness (0–100)			
Raw mean ± SD	29.2 ± 24.3	27.0 ± 23.2	27.8 ± 23.6
EMM ± SE	33.9 ± 3.3	31.7 ± 3.3	32.5 ± 3.3
EMM 95% CI	27.3–40.6	25.1–38.4	25.9–39.1
Median (IQR)	22.7 (9.0–51.0)	23.5 (8.2–42.5)	22.3 (8.7–41.9)

* Lower value after post hoc pairwise comparison relative to other stimulation sites, *P* < 0.001.

† Higher value after post hoc pairwise comparison relative to eyelid, *P* < 0.001.

‡ Higher value after post hoc pairwise comparison relative to forehead, *P* < 0.05.

CI, confidence interval (lower bound–upper bound); EMM, estimated marginal means; IQR, interquartile range.

**Figure 3.**
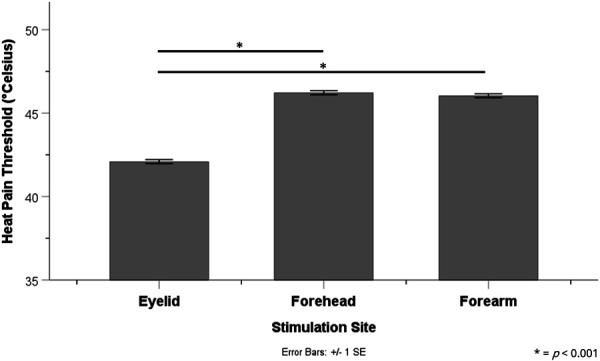
Mean HPT for the eyelid, forehead, and forearm. Post hoc pairwise comparisons show significantly lower eyelid HPT relative to other stimulation sites.

Linear mixed model analysis showed a significant effect of stimulation site on HPT (*P* < 0.001), and post hoc pairwise analysis with Bonferroni correction revealed significantly lower HPT at the eyelid (42.5 ± 0.4°C) compared with the forehead (46.6 ± 0.4°C) (*P* < 0.001) and the forearm (46.4 ± 0.4°C) (*P* < 0.001). Heat pain threshold was not significantly different between the forehead and forearm (*P* = 1.00) (Fig. 3).

Linear mixed model further showed that sex (45.6 ± 0.5°C male vs 44.8 ± 0.5°C female; *P* = 0.20) and study site (44.5 ± 0.3°C BCH vs 45.9 ± 0.7°C UMiami; *P* = 0.12) did not have a significant impact on HPT, while age (PE = 0.06 ± 0.03, *P* = 0.067) displayed a trend towards, but did not reach, statistical significance.

### 4.3. Pain intensity and unpleasantness visual analogue scale ratings at heat pain thresholds for the eyelid, forehead, and forearm

Mean and median pain intensity and unpleasantness ratings for each stimulation site were averaged across subjects. Estimated marginal means were calculated to adjust for age as a covariate. Age was evaluated at 25.1 years to prevent age differences from affecting other factor analyses (Table 2).

Linear mixed model analysis revealed a significant effect of stimulation site on pain intensity ratings (*P* < 0.001), and post hoc analysis with Bonferroni correction showed that despite the eyelid having a lower HPT, pain intensity ratings on the forearm (26.9 ± 3.1) were significantly higher than on the eyelid (24.0 ± 3.1) (*P* < 0.001) and on the forehead (24.8 ± 3.1) (*P* = 0.015), with no significant difference between the eyelid and forehead (*P* = 0.79). Unlike pain intensity ratings, LMM analysis did not show a significant effect of stimulation site (*P* = 0.11) on unpleasantness ratings, which was confirmed by Bonferroni-corrected post hoc analysis between the eyelid (33.9 ± 3.3) and forehead (31.7 ± 3.3) (*P* = 0.12), the eyelid and forearm (32.5 ± 3.3) (*P* = 0.53), and the forehead and forearm (*P* = 1.00).

Linear mixed model showed that sex had no effect on pain intensity (23.5 ± 3.8 male vs 27.1 ± 3.9 female; *P* = 0.43) or unpleasantness (32.9 ± 4.1 male vs 32.6 ± 4.2 female; *P* = 0.96) ratings.

Although pain intensity ratings were statistically equivalent between sites (24.2 ± 2.7 BCH vs 26.3 ± 5.8 UMiami; *P* = 0.75), LMM analysis revealed a significant effect of study site (24.8 ± 2.9 BCH vs 40.6 ± 6.2 UMiami; *P* = 0.03) on unpleasantness ratings.

Interestingly, age had a significant positive impact on pain intensity ratings, wherein increasing age was associated with an increase in pain intensity ratings at threshold (PE = 0.52 ± 0.25, *P* = 0.040), but had no significant effect on unpleasantness ratings (PE = 0.23 ± 0.27, *P* = 0.40).

## 5. Discussion

In this study, we compared HPTs assessed by the ascending method of limits at 3 stimulation sites: the eyelid, the forehead, and the forearm. Heat pain threshold at the eyelid was significantly lower than HPT at the forehead and forearm in healthy participants. Heat pain threshold differed between the eyelid and the other stimulation sites despite all being classified as hairy skin,^46,49^ and also differed from the forehead despite both stimulation sites receiving innervation from the supraorbital nerve, a branch of the ophthalmic division of the trigeminal nerve.^8,30,35^ This is similar to QST assessments performed elsewhere in the body that have revealed variations in thermal sensitivity in areas innervated by separate nerves as well as by subdivisions of the same nerve.^40,50,64^ Previous reports have identified differential HPTs in areas innervated by the maxillary division of the trigeminal nerve, where the most sensitive sites were found to be the lip area (HPT: 42.3–42.9°C) and the infraorbital cheek (HPT: 41.0°C).^17,50^ These temperatures are similar to the HPTs measured on the eyelid in this study (42.0°C). However, the stimulation area in the current study (28.27 mm^2^) is much smaller than that used to evaluate the lip (96.8 mm^2^) and the infraorbital cheek (81.0 mm^2^), preventing a direct comparison.^15,17,50^

Several factors may contribute to lower HPTs on the eyelid when compared with the forehead and forearm, including differences in afferent innervation. Although both the eyelid and the forehead stimulation sites are innervated by the supraorbital nerve, the eyelid features additional innervation from multiple subdivisions of the frontal nerve (supraorbital, supratrochlear) and a branch of the nasociliary nerve (infratrochlear).^30,32,43^ The cumulative thermal inputs from multiple nociceptors of the eyelid may converge to trigger a pain sensation, a phenomenon related to spatial summation.^51,53,61^ In experimental settings, spatial summation of heat pain is often a factor when comparing stimuli delivered by different size thermodes. However, the density and architecture of eyelid innervation may contribute to spatial summation in this case, as larger numbers of primary nerve afferents may be activated relative to the forehead.

Another possibility may be the involvement of corneal afferents. The skin on the eyelid is among the thinnest in the human body,^10,39^ which could allow thermal stimuli to penetrate the eyelid and affect receptors on the cornea. We cannot determine from these data whether the perceived sensations were appreciated by the eyelid alone or whether corneal sensation influenced HPT values. Notably, some participants recorded experiencing heat pain at temperatures as low as 36°C, which align closely with temperatures that can elicit pain sensations in the cornea (37–39°C), attributed to activation of polymodal nociceptors.^1^ Some participants in this study, however, reported HPTs as high as 49°C on the eyelid, and so this difference may be the result of intersubject variability in pain sensitivity rather than recruitment of corneal thermoreceptors.^12,13^

Another potential source of intersubject variability is lid thickness, which may affect the involvement of corneal afferents and contribute to the wide, bimodal distribution of HPT values observed with eyelid testing (Fig. 2). Mean facial skin thickness in the lower forehead is approximately 1,222 ± 343 µm, whereas the upper medial eyelid has a mean thickness of 799 ± 445 µm, highlighting lid thinness as well as the significant interindividual variability in eyelid structure.^10^ It may be that thinner lids are associated with greater heat transfer to the cornea and thus a lower HPT, while thicker eyelids have higher HPTs because of less corneal involvement. However, this study cannot comment on this possible phenomenon, which would require measurement of eyelid thickness to further delineate its contribution to HPT.

Isolation of the cornea and heating of the eyelid has not been thoroughly examined experimentally; clinically, however, it is routinely performed as part of thermodynamic treatments for Meibomian gland dysfunction and dry eye disease.^63,71^ These clinical treatments provide some insight regarding sensations evoked by thermal stimulation to the eyelids, without contributions from corneal afferents. The LipiFlow System heats the eyelids to between 41 to 43°C and is designed to avoid corneal contact or stimulation.^19^ Another device, the iLux MGD treatment system, works similarly and regulates eyelid heating to not exceed 45°C.^63^ Despite the temperatures reached by the devices, which our results indicate should reliably cause thermal pain, the treatment is well tolerated and only 2/377 participants in comparative studies reported burning sensations.^63,71^ Topical numbing drops are applied on the cornea before using these devices; however, this is insufficient to block eyelid sensation as evidenced by previous investigation and the fact that eyelid tenderness assessments are performed after topical corneal anesthesia.^7,70^ Thus, when the cornea is isolated, the eyelids routinely tolerate temperatures exceeding HPT values reported in this study, supporting the potential engagement of corneal afferents when thermal stimuli are applied to the closed upper eyelids.

Interestingly, despite findings of lower HPTs on the eyelid, pain intensity ratings were higher at the forearm than the eyelid or forehead. The variation in pain intensity ratings between stimulation sites may reflect differences in receptor density, underlying nerve fiber types, or differential signaling through neuronal circuits and brain regions underlying distinct dimensions of pain, resulting in a fundamentally different pain experience despite identical stimulation.^33,34^ However, the difference in pain intensity ratings was <3/100 and thus is unlikely to be clinically meaningful.

Although no difference was found in VAS unpleasantness ratings between stimulation sites, we found a significant difference between study sites. Differences in demographics or other factors between study sites may underlie these findings; however, this cannot be fully investigated here, given power limitations. Notably, pain intensity ratings were not affected in the same way, which may imply the influence of factors outside the execution of the protocol as this suggests participants were indicating the first presence of a pain sensation uniformly across study sites.

Age was significantly correlated with pain intensity ratings, but not with HPT or unpleasantness ratings. There is evidence that pain ratings are elevated in older participants, particularly for stimuli 49°C and above.^27^ However, many studies describe reductions in sensitivity across most QST methods with increasing age.^26,54,72^ Others report variable, weak age trends that are inconsistent across modalities.^36,48^ The effect of age on pain sensitivity is most studied in those age 40 years and older,^23,48,54^ and is most pronounced in those age 60 years and older for low-intensity heat pain stimuli.^37^ Thus, the influence of age may not have been well characterized by our youth-weighted cohort, given the disparities between the relationship of age and HPT, pain intensity, and unpleasantness described here.

We found no effect of sex on HPT or VAS ratings. There are contradictory reports in the literature describing whether a difference in pain sensitivity between sexes exists, and this topic is still an active area of research.^45,52,59^ Notably, there is evidence that sex has a comparatively small effect on HPT relative to other QST modalities,^48^ which may explain why we did not observe sex differences in our data.

Our study has several strengths, including a relatively large sample size (n = 80), diverse age range, and replication at 2 study sites, supporting the finding of meaningful difference in HPT across the stimulation sites tested. However, the study also has limitations. A large concentration of our participants falls within the young adult demographic; a more evenly distributed sample in future studies would allow the influence of age to be more thoroughly explored and allow for better comparison to patients with COP. Furthermore, as noted above, the stimuli administered to the eyelid may have activated sensory nerves in the cornea, and it is impossible to determine the extent of corneal involvement in eyelid HPT from these data. Previous investigations have attempted to disentangle eyelid and cornea sensations using a physical barrier (eg, contact lens) to block thermal transmission to the cornea or topical anesthetic to isolate sensory transmission from the lid,^7^ and some version of these methodologies could be used in future studies. This study used ramping stimuli to assess HPTs, and anxiety and avoidance behaviors can influence threshold results in method of limits testing.^24^ Although there were no significant differences in pain intensity ratings between the forehead and eyelid, suggesting that avoidance behavior did not result in a lessened pain experience, that does not rule out the contribution of anxiety or fear to an enhanced pain experience. Future studies should collect anxiety and fear of pain questionnaire data to explore any relationship these psychological factors may have on HPTs as well as pain intensity and unpleasantness ratings at the eyelid, compared with other test site locations. Another limitation is that HPT assessment order was not randomized, thus, an order or familiarity effect could have influenced the data. To mitigate this potential effect, a practice test was given before testing began and assessments were performed and repeated in sequence (forehead, then forearm, then eyelid, repeated 3 times) rather than performing repetitions at each site independently (3 trials at forehead, then 3 trials at forearm, then 3 trials at eyelid). Future studies should endeavor to assess each site independently or to vary the order of presentation. Given the small surface area of the eyelid, this study required a smaller thermode (28.3 mm^2^) than is commonly used in other QST protocols (900 mm^2^). Larger thermode contact area can evoke a spatial summation effect, resulting in lower thresholds and increased temperature sensitivity.^15,50^ The smaller probe used here may have resulted in some participants reaching the 50.1°C safety cutoff temperature before discerning pain, artificially capping HPT and inducing a ceiling effect on the data (as can be appreciated in the forehead and forearm HPT distributions in Fig. 2). In addition, the thermode was placed on each stimulation site by hand, and while the use of consistent pressure is emphasized in the protocol, small differences in pressure with manual application could have influenced subjective experiences during testing. Finally, there was an uneven sample size between study sites, and so the statistical equivalency between sites is less compelling than it would be with matched participant numbers. Future research should specifically address questions regarding site uniformity by prospectively matching sample size and demographics between sites. Future studies could expand upon our work by incorporating other thermal testing paradigms (eg, method of constant stimuli) and temperatures (eg, cold pain), as well as other pain modalities (eg, mechanical), and aim to uncover the underlying physiology that contributes to the bimodal nature of eyelid HPT distributions.

Our goal is to develop personalized treatment regimens^3,18,42^ for individuals with ocular pain, and personalized therapies require precise mechanistic diagnoses for each patient. Chronic ocular pain can have multiple contributors, and different etiologies can present similar symptoms. We believe some causes of COP are restricted to peripheral nerves and receptors in the eye or ophthalmic branch of the trigeminal nerve, while others are the result of a more systemic, centralized dysfunction^11,22,57^; however, many manifestations of COP can have varying contributions from all of these sources depending on the patient.^5,9,56^ The ability to contrast localized eyelid sensory profiles with extra-ocular sites using QST could allow investigators to interrogate differences in the contributions of peripheral vs centralized nerve disruption in each patient—fingerprinting the precise balance of mechanisms in an individual that underlie their pain. This individualized phenotyping may be useful in unraveling the puzzling, often overlapping etiologies present in COP, and the classification of relevant disease subtypes. This could also provide a method to create bespoke therapy for each patient, rather than attempting to ameliorate a generalized constellation of symptoms. The HPTs described here are an initial foray into the investigation of eyelid sensory profiles, which could prove valuable in the creation and implementation of standardized ocular somatosensory assessments that may aid in the diagnosis and management of ocular pain conditions.

## Disclosures

The authors have no conflict of interest to declare.

## Supplemental digital content

Supplemental digital content associated with this article can be found online at http://links.lww.com/PR9/A344.

## Supplementary Material

SUPPLEMENTARY MATERIAL
