# Supplementary material for: Eyelid heat pain sensitivity in healthy participants
Source: Pain Rep. 2025 Sep 22;10(5):e1337. doi: 10.1097/PR9.0000000000001337 (PMC12456492; doi:10.1097/PR9.0000000000001337)
Supplement: SUPPLEMENTARY MATERIAL [file painreports-10-e1337-s001.pdf]

<u>Supplementary Methods:</u>

Supplementary QST Analysis- Full Script:

“We are going to measure your sensitivity, or threshold, for hot pain. The probe will start out at about the same temperature as your skin, then the temperature will increase to ‘warm’ and a few moments later to ‘hot.’ Eventually a painful component will be added to the sensation of ‘hot’ and it will change in quality from ‘hot’ to, for example, ‘burning’ or ‘stinging hot.’

Please press the stop-button immediately at the first ‘burning’ or ‘stinging’ hot sensation

After the trial I’ll show you a line that represents the entire continuum of pain intensity, with “no pain at all” on the left side and “the most intense pain imaginable” on the right side. I’ll ask you to mark the intensity of the pain you felt at the time you pressed the button on that line. Please make sure that you are rating the intensity of the pain, not how hot the stimulus felt.”

“Then I’ll show you another line and ask you to mark the unpleasantness of the sensation you felt at the time you pushed the button. On the left side is “not unpleasant at all” and the right side is “the most unpleasant sensation imaginable.”

Do you have any questions about this?

First, we’ll do a practice trial on your hand, so you will know what to expect, and you can practice rating the sensation you felt.”

“Now we will perform 3 trials on each test site. We will start with trial 1 on your forehead, then do 1 on your forearm, then 1 on your eyelid. Then we will repeat this 2 more times at each test site. There will be about 30-45 seconds between each trial.”

Supplementary Data Analysis:

To assess the reliability of HPT data, the Intra-class Correlation Coefficient was performed for each stimulation site.

The number of subjects was large enough to allow the use of parametric analyses regardless of potential violation of assumptions, however, as eyelid QST has not been widely studied we assessed these metrics regardless. Shapiro-Wilk tests were performed to check for normality of distribution in HPT, pain intensity, and unpleasantness data for each stimulation site.

To confirm parametric LMM analysis results, a related-Samples Friedman’s Two-Way Analysis of Variance by Ranks test was performed in instances where violations of the parametric assumption of normality were found. Post-hoc pairwise comparisons were performed and significance values were adjusted by the Bonferroni correction to account for multiple test comparisons. Values for HPT, pain intensity, and unpleasantness variables are reported as Median [Interquartile Range (IQR)]. A p value of 0.05 or less was considered statistically significant.

Statistical analyses were performed using SPSS software ver. 29 (IBM, Armonk, NY).

Supplementary Results:

Participant comorbidities and medications are presented in **Supplementary Table 1**. Medications taken by participants were stable and taken for ≥4 weeks. While not linked with alteration of pain perception, starting or stopping some of the included medications (e.g., systemic birth control, allergy medicine) can affect mood or inflammatory background, and so in turn affect psychometric data.

Supplementary Table 1: Comorbidities and Medications

	**Medication (Yes/No)**	**n**
Acne	Yes	3
ADHD	Yes	1
Anxiety/Depression	Yes	4
Environmental allergy	Yes	5
Birth control	Yes	8
GERD	Yes	1
Hair loss	Yes	1
HIV prophylaxis	Yes	1
Iron deficiency	Yes	1
Puberty blocker	Yes	1
Antibiotic	Yes	1
Vitiligo	No	1

ADHD = attention deficit hyperactive disorder, GERD = gastroesophageal reflux disease, HIV = Human Immunodeficiency Virus

Participant ICCs revealed excellent intra-observer reliability of HPT and are shown in **Supplementary Table 2**.

Supplementary Table 2: HPT Intra-class Correlation Coefficient

Stimulation Site	Intraclass Correlation	95% CI Lower	95% CI Upper	Value	df1	df2	Sig
Eyelid	0.94	0.92	0.96	17.85	78	156	<0.001
Forehead	0.92	0.88	0.95	12.10	78	156	<0.001
Forearm	0.94	0.91	0.96	16.55	78	156	<0.001

95% CI Lower = Confidence Interval Lower Bound
95% CI Upper = Confidence Interval Upper Bound
df = degrees of freedom
Sig. = statistical significance

Full HPT LMM results and data are displayed in **Supplementary Table 3**.

Supplementary Table 3: HPT Repeated Measures Linear Mixed Model Estimates of Fixed Effects

Parameter	Estimate	SE	df	t	Sig.	95% CI Lower	95% CI Upper
Intercept	41.30	1.37	77.87	30.09	<0.001	38.56	44.03
Eyelid	4.13	0.26	158.0	15.80	<0.001	3.62	4.65
Forehead	3.95	0.26	158.0	15.11	<0.001	3.43	4.47
Forearm	0[b]	0					
Male	0.76	0.59	76.0	1.30	0.20	-0.41	1.93
Female	0 [b]	0					
BCH	-1.33	0.85	76.0	-1.58	0.12	-3.02	0.35
UMiami	0 [b]	0					
Age	0.06	0.03	76.0	1.86	0.067	-0.004	0.12

[b] = Parameter set to 0 because it is redundant
SE = Standard Error
df = degrees of freedome
t = t statistic
Sig. = statistical significance
95% CI Lower = Confidence Interval Lower Bound
95% CI Upper = Confidence Interval Upper Bound

Shapiro-Wilks tests indicated data for HPT, pain intensity ratings, and unpleasantness ratings at all stimulation sites were not normally distributed **(Supplementary Table 4)**.

Supplementary Table 4: Summary of Shapiro-Wilks normality of distribution tests.

	**Statistic**	**df**	***p***
**HPT**			
Eyelid	0.951	80	0.004
Forehead	0.901	80	<0.001
Forearm	0.946	80	0.002
**Pain Intensity**			
Eyelid	0.866	80	<0.001
Forehead	0.882	80	<0.001
Forearm	0.897	80	<0.001
**Unpleasantness**			
Eyelid	0.899	80	<0.001
Forehead	0.902	80	<0.001
Forearm	0.909	80	<0.001

df = Degrees of Freedom

Non-parametric HPT analyses for the eyelid, forehead, and forearm

Median data for each stimulation site was averaged across subjects for HPTs **(Supplementary Table 5)**.

Supplementary Table 5: Median HPT, pain intensity, and unpleasantness

	Eyelid	Forehead	Forearm
**HPT (°C)**			
Median [IQR]	**42.1[38.8-45.5]***	46.7[44.4-48.3]	46.5[44.7-48.0]
**Pain Intensity (0-100)**			
Median [IQR]	18.3[6.1-32.8]	16.5[7.7-36.8]	**22.2[8.9-37.0]**$^{+\wedge}$
**Unpleasantness (0-100)**			
Median [IQR]	22.7[9.0-51.0]	23.5[8.2-42.5]	22.3[8.7-41.9]

IQR = Interquartile Range
* = lower value after post-hoc pairwise comparison relative to other stimulation sites, $p<0.001$.
+ = higher value after post-hoc pairwise comparison relative to the eyelid, $p<0.001$
^ = higher value after post-hoc pairwise comparison relative to forehead, $p<0.05$

A Friedman test supported the LMM findings and revealed a significant difference between the distributions of HPTs among stimulation sites ($p<0.001$), with Bonferroni-corrected post-hoc pairwise analysis showing significant differences in median HPT between the eyelid (42.1[38.8-45.5] °C) and forehead (46.7[44.4-48.3]°C) ($p<0.001$) as well as the eyelid and forearm (46.5[44.7-48.0] °C) ($p<0.001$), but not between the forehead and forearm (p=1.00).

Non-parametric pain intensity and unpleasantness VAS ratings at HPT for the eyelid, forehead, and forearm

Median pain intensity and unpleasantness ratings for each stimulation site were averaged across subjects **(Supplementary Table 5)**.

A Friedman test confirmed significantly different pain intensity ratings between stimulation sites ($p<0.001$). Post-hoc analysis showed pain intensity ratings from the forearm (22.2[8.9-37.0]) were significantly greater than the eyelid (18.3[6.1-32.8]) ($p<0.001$) as well as from the forehead (16.5[7.7-36.8]) ($p=0.005$), but no difference was found between the eyelid and forehead ($p=0.969$).

A Friedman test revealed no significant difference in unpleasantness ratings between stimulation sites ($p=0.098$), which was further confirmed with post-hoc pairwise analysis with Bonferroni correction between the eyelid (22.7[9.0-51.0]) and forehead (23.5[8.2-42.5]) ($p=0.158$), the eyelid and forearm (22.3[8.7-41.9]) ($p=1.00$), or forehead and forearm ($p=0.315$).
